# Effects of channel length on temperature dependence of apparent subthreshold swing in self-aligned top-gate coplanar IGZO thin-film transistors

**DOI:** 10.1038/s41598-025-18234-0

**Published:** 2025-10-06

**Authors:** Chae-Eun Oh, Hwan-Seok Jeong, Su-Hyeon Lee, Hyeon-Woo Lee, Dong-Hwi Son, Chang-Hyeon Kim, Myeong-Ho Kim, Kyoung-Seok Son, Jun Hyung Lim, Sang-Hun Song, Hyuck-In Kwon

**Affiliations:** 1 Major in Intelligent Semiconductor Engineering, Chung-Ang University, 84 Heukseok-ro, Dongjak-gu, Seoul, Korea; 2 School of Electrical and Electronics Engineering, Chung-Ang University, 84 Heukseok-ro, Dongjak-gu, Seoul, Korea; 3 Samsung Display, 162, Nongseo-ro, Giheung-gu, Yongin-si, Gyeonggi-do Korea

**Keywords:** SA TG coplanar IGZO TFT, Temperature, Apparent subthreshold swing, Channel length, Stability, Electronic devices, Electrical and electronic engineering

## Abstract

This study investigates how channel length (*L*) affects the temperature dependence of the apparent subthreshold swing (SS^*^) in self-aligned top-gate coplanar indium-gallium-zinc oxide (IGZO) thin-film transistors (TFTs). Our experimental results demonstrate that SS^*^ increases with temperature for devices with *L* = 5 μm and 7 μm, yet decreases for devices with *L* = 10 μm. To elucidate this behavior, we developed a drain current model based on surface potential. Our analysis reveals that variations in the dominant carrier populations (trapped versus free electrons) within the SS^*^ extraction range dictate the observed temperature dependence of SS^*^. Furthermore, the lateral diffusion of donor impurities in our fabricated structures leads to notable differences in the effective channel length across TFTs with varying *L*s, thereby amplifying these trends. These findings offer crucial insights into the physical mechanisms underlying the channel-length-dependent temperature behavior of SS^*^ in IGZO TFTs. This understanding is vital for enhancing the stability of active-matrix organic light-emitting diode displays that utilize IGZO TFTs as backplanes.

## Introduction

Indium–gallium–zinc oxide (IGZO) is a suitable replacement material for amorphous silicon (a-Si) and has been adopted in thin-film transistors (TFTs) owing to its high carrier mobility, low process temperature, and excellent large-area uniformity. Accordingly, IGZO TFTs are currently used in commercial display products such as active-matrix organic light-emitting diode (AMOLED) displays^1,2^. For high image quality in AMOLED displays, the pixel circuits in the display panel must maintain a constant OLED current because the OLED emits light with an intensity proportional to the OLED current. However, variations in the electrical properties of the driving TFTs degrade the image quality of AMOLED displays^3^. In particular, variation in the subthreshold swing (*SS*) of the driving TFTs degrades image quality at low gray levels in AMOLED displays^4,5^. Because the human eye is more sensitive to image degradation at low gray levels than at high gray levels^6^, it is very important to study the effects of operating conditions on the *SS* values of driving TFTs in AMOLED displays. Among various operating conditions, operating temperature has been reported to have a strong influence on the *SS* values of oxide TFTs^7–9^. However, previous studies have reported inconsistent experimental results regarding the temperature dependence of *SS* values in oxide TFTs. Most research groups reported an increase in *SS* values with an increase in operating temperature from 300 to 360 K^10,11^, whereas a few groups reported a decrease in *SS* values with increasing temperature in a similar temperature range^12,13^. Unfortunately, the physical mechanism responsible for these inconsistent results has not yet been identified. Because AMOLED displays operate across a wide range of temperatures, it is crucial to systematically study the temperature dependence of *SS* values in IGZO TFTs so that consistent image quality can be maintained over a wide temperature range with appropriate compensation methods. In this study, we observed that the channel length (*L*) of the device can significantly affect the temperature dependence of the apparent *SS* (SS^*^) in the fabricated IGZO TFTs, where SS^*^ was defined as the gate voltage (*V*_G_) required to increase the drain current (*I*_D_) from 10^−12^ A to 10^−11^ A at a drain voltage (*V*_D_) of 0.1 V, based on the current range commonly used to extract *SS* in several previous studies on IGZO TFTs including Refs.^14,15^. This current range corresponds to the operating current level of AMOLED pixels under low-luminance conditions. To elucidate the origin of this behavior, an *I*_D_ model based on the surface potential (*φ*_S_) was derived for IGZO TFTs. The development of compact models based on mathematical formulations has been shown to be valuable in describing complex system characteristics^16,17^. By comparing the calculated and measured *I*_D_ characteristics at various temperatures for devices with different *L*s, the underlying mechanism responsible for the observed phenomena was identified.

## Results and discussion

**Fig. 1:**
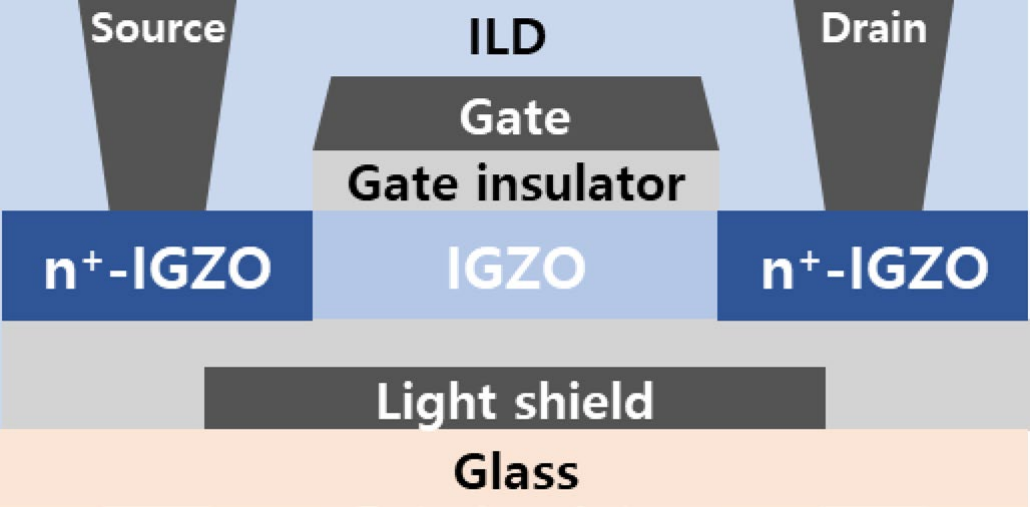
Schematic cross-section of the fabricated SA TG coplanar IGZO TFTs.

**Fig. 2:**
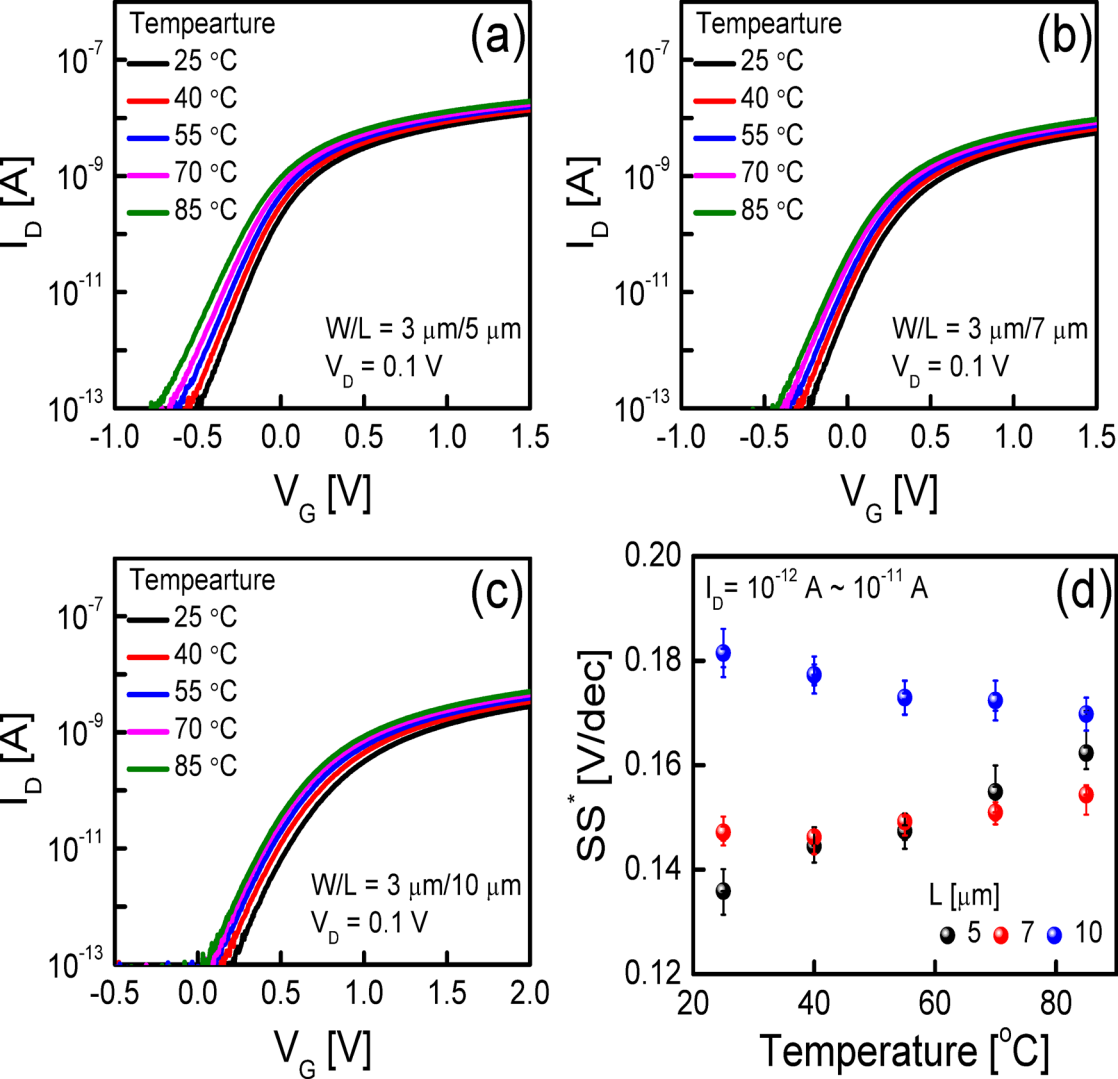
Representative transfer characteristics of the fabricated SA TG coplanar IGZO TFTs with *L* of (**a**) 5 μm, (**b**) 7 μm, and (**c**) 10 μm, measured at different temperatures (25–85 °C) at *V*_D_ = 0.1 V. The *W* was fixed at 3 μm in all devices. (**d**) SS^*^ values extracted from the fabricated IGZO TFTs with *L* of 5 μm, 7 μm, and 10 μm at different temperatures, where each value represents the average of five devices, and the vertical error bars indicate ± 1 standard deviation.

Figure 1 illustrates a schematic cross-section of the fabricated self-aligned top-gate (SA TG) coplanar IGZO TFT. The fabrication process is described in detail in the Methods section. The SA TG coplanar structure is commonly employed in the fabrication of IGZO TFTs for AMOLED applications due to its low parasitic capacitance and excellent process control^18,19^. Figures 2a–c show the transfer characteristics (*V*_G_ versus *I*_D_) of the fabricated SA TG coplanar IGZO TFTs measured at temperatures from 25 °C to 85 °C with *V*_D_ = 0.1 V. The *L* was set to (a) 5 μm, (b) 7 μm, and (c) 10 μm, respectively, with the channel width (*W*) fixed at 3 μm. Transfer curves in Figs. 2a–c are taken from representative devices among five measured samples for each *L*. All electrical measurements were performed using an Agilent 4156C semiconductor parameter analyzer, with the TFT’s source and back gate electrically connected. Temperature-dependent measurements were conducted using a temperature-controlled thermal chuck system, where the devices were allowed to stabilize at each target temperature for at least 5 min before data acquisition. All measurements were performed in the dark. Figures 2a–c show that the transfer curve shifts negatively with a decrease in *L*. These results are consistent with those in the previous works and the more negative shift of the transfer curves in shorter-channel TFTs was attributed to the higher carrier concentration in the channel region caused by the carrier diffusion from the n^+^-doped source/drain extension regions^18^. Figure 2d summarizes the SS^*^ values extracted as the inverse slope of the transfer curve (d*V*_G_/d(log *I*_D_)) over the range of 10⁻^12^ A < *I*_D_ < 10⁻^11^ A, following definitions used in prior studies on IGZO TFTs. The plotted values represent averages over five devices for each *L*, with vertical error bars indicating ± 1 standard deviation. Figure 2d shows that the average SS^*^ value obtained from the IGZO TFTs with *L* = 5 and 7 μm increases with an increase in the temperature from 25 to 85 °C (from 0.137 to 0.164 V/decade for TFT with *L* = 5 μm and from 0.144 to 0.152 V/decade for TFT with *L* = 7 μm), whereas that obtained from the IGZO TFT with *L* = 10 μm slightly decreases from 0.181 to 0.172 V/decade with an increase in the temperature from 25 to 85 °C. The results Fig. 2d clearly show that the channel length of the device strongly affects the temperature dependence of SS^*^ in the fabricated SA TG coplanar IGZO TFTs.

**Fig. 3:**
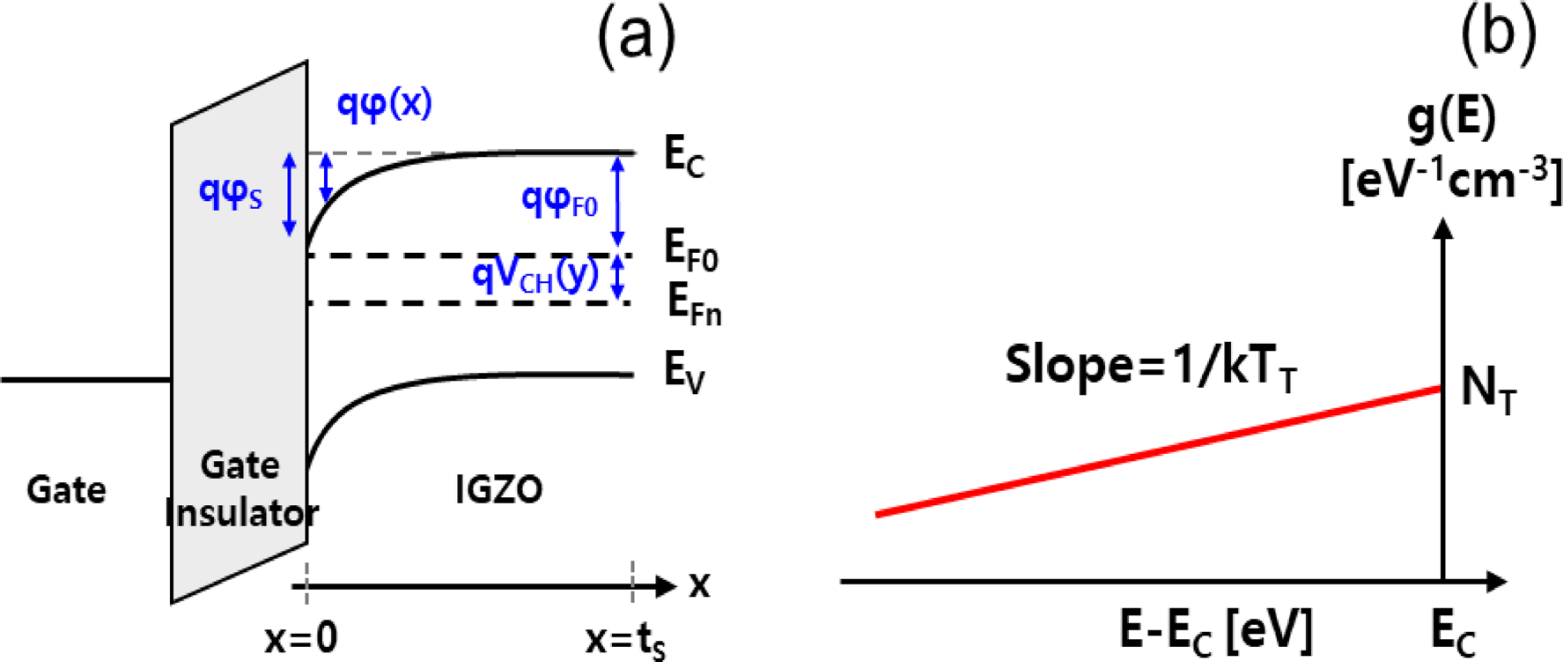
(**a**) Energy band diagram along the thin-film depth direction of the SA TG coplanar IGZO TFT. (**b**) Schematic illustration of the subgap density of acceptor-like states over the bandgap near *E*_C_ (*g*(*E*)) in IGZO.

To elucidate the origin of this behavior, an *I*_D_ model based on *φ*_S_ was developed for IGZO TFTs. Figure 3a illustrates the energy band diagram along the depth direction of the IGZO thin film. In the diagram, *q* is the elementary charge, *t*_S_ is the IGZO film thickness, x is the position along the vertical direction measured from the IGZO/gate insulator interface, y is the coordinate along the lateral direction from the source, *φ*_F0_ is the bulk-Fermi potential, *φ*(x) is the electric potential along the depth direction, *E*_F0_ is the Fermi level at the flat-band condition, *E*_C_ is the conduction band minimum, *E*_V_ is the valence band maximum, *V*_CH_(y) is the potential difference [i.e., describing the electron quasi-Fermi level *E*_Fn_ lowered by *qV*_CH_(y) due drain voltage *V*_D_ along the channel length direction]. In this study, the subgap states near *E*_C_ are assumed to consist of the acceptor-like trap states that are exponentially distributed in energy in IGZO^12,13^. These trap states originate from structural disorder in the amorphous IGZO matrix, such as variations in metal–oxygen bonding and oxygen vacancy-related defects, which create localized states near *E*_C_^20,21^. Figure 3b shows a schematic illustration of the subgap density of acceptor-like states over the bandgap near *E*_C_ (*g*(*E*)) in the model^22^:

1 $$g\left( E \right) = N_{T} \exp \left( {\frac{{E - E_{C} }}{{kT_{T} }}} \right),$$

where *E* is the electron energy, *N*_T_ is the density of trap states extrapolated to *E*_C_, *k* is the Boltzmann constant, and *kT*_T_ is the characteristic energy of the acceptor-like states. Based on the gradual channel approximation, the one-dimensional Poisson equation along the channel depth (*x*) can be expressed as^23^

2 $$\frac{{d^{2} \varphi \left( x \right)}}{{dx^{2} }} = \frac{q}{{\varepsilon_{S} }}\left( {n_{Free} \left( x \right) + n_{Trap} \left( x \right)} \right)\,,$$

where *ε*_S_ is the IGZO permittivity. Here, *n*_Trap_(*x*) and *n*_Free_(*x*) represent the densities of trapped electrons in localized acceptor-like trap states and free electrons, respectively, and can be expressed as^24,25^

3 $$n_{Trap} \left( x \right) \approx N_{T} kT_{T} \exp \left[ {\frac{{E_{F0} - E_{C} + q\varphi \left( x \right) - qV_{CH} }}{{kT_{T} }}} \right],$$

4 $$n_{Free} (x) \approx N_{C} \exp \left[ {\frac{{E_{F0} - E_{C} + q\varphi \left( x \right) - qV_{CH} }}{kT}} \right]\,,$$

where *N*_C_ is the effective density of states of free electrons and *T* is the temperature. Then, the electric field inside the IGZO thin film (*E*_IGZO_(*x*)), which is defined as Eq. (5),

5 $$E_{IGZO} \left( x \right) = - \frac{d\varphi \left( x \right)}{{dx}}$$

can be solved by multiplying both sides of Eq. (2) by 2*φ*(*x*)/d*x* and then integrating over *x*. Together with the boundary conditions *φ*(*t*_S_) = d*φ*(*t*_S_)/d*x* = 0, d*φ*(*x*)/d*x* can be solved as Eq. (6)^11^.

6 $$\begin{gathered} \int\limits_{{t_{S} }}^{x} {2\frac{d\varphi \left( x \right)}{{dx}} \cdot \frac{{d^{2} \varphi \left( x \right)}}{{dx^{2} }}dx} = \int\limits_{{t_{S} }}^{x} {\frac{d}{dx}\left( {\frac{d\varphi \left( x \right)}{{dx}}} \right)^{2} dx} = \left( {\frac{d\varphi \left( x \right)}{{dx}}} \right)^{2} \hfill \\ \int\limits_{{t_{S} }}^{x} {\frac{2q}{{\varepsilon_{S} }}\left( {\frac{d\varphi \left( x \right)}{{dx}}} \right)\left[ {n_{Free} \left( x \right) + n_{Trap} \left( x \right)} \right]dx} = \frac{2q}{{\varepsilon_{S} }}\int\limits_{0}^{\varphi \left( x \right)} {\left[ {n_{Free} \left( x \right) + n_{Trap} \left( x \right)} \right]d\varphi \left( x \right)} \hfill \\ \Rightarrow E_{IGZO} \left( x \right) = - \frac{d\varphi \left( x \right)}{{dx}} = \sqrt {\frac{2q}{{\varepsilon_{S} }}\int\limits_{0}^{\varphi \left( x \right)} {\left[ {n_{Free} \left( x \right) + n_{Trap} \left( x \right)} \right]d\varphi \left( x \right)} } \hfill \\ \end{gathered}$$

By using Eq. (5) and substituting Eqs. (3) and (4) into Eq. (6), the surface electric field *E*_S_ = *E*_IGZO_(*x* = 0) can be expressed as

7 $$\begin{aligned} E_{S} & = \sqrt {\frac{{2q}}{{\varepsilon _{S} }}\int\limits_{0}^{{\varphi _{S} }} {\left[ {n_{{Free}} \left( x \right) + n_{{Trap}} \left( x \right)} \right]d\varphi \left( x \right)} } \\ & = \sqrt {\frac{{2N_{T} \left( {kT_{T} } \right)^{2} }}{{\varepsilon _{S} }}\exp \left( {\frac{{E_{{F0}} - E_{C} + q\varphi _{s} - qV_{{CH}} }}{{kT_{T} }}} \right) + \frac{{2N_{C} kT}}{{\varepsilon _{S} }}\exp \left( {\frac{{E_{{F0}} - E_{C} + q\varphi _{s} - qV_{{CH}} }}{{kT}}} \right)} \\ \end{aligned}$$

Using Gauss’s law with Eq. (7),

8 $$\begin{aligned} \varepsilon _{S} E_{S} & = C_{{OX}} \left( {V_{G} - V_{{FB}} - \varphi _{S} - V_{{CH}} } \right) \\ & = \sqrt {2\varepsilon _{S} N_{T} \left( {kT_{T} } \right)^{2} \exp \left( {\frac{{E_{{F0}} - E_{C} + q\varphi _{s} - qV_{{CH}} }}{{kT_{T} }}} \right) + 2\varepsilon _{S} N_{C} kT\exp \left( {\frac{{E_{{F0}} - E_{C} + q\varphi _{s} - qV_{{CH}} }}{{kT}}} \right)} , \\ \end{aligned}$$

where *V*_FB_ and *C*_OX_ denote the flat-band voltage and gate insulator capacitance per unit area, respectively. From Eq. (8), *φ*_S_ can be obtained as a function of *V*_G_.

Most acceptor-like trap states below *E*_Fn_ are occupied by electrons at low temperatures (*kT *≈ 0 K) in IGZO; however, electrons can be released by thermal excitation from the occupied trap states into the conduction band and can contribute to *I*_D_ with an increase in temperature^26,27^. In IGZO TFTs, at low *V*_G_s, *n*_Trap_(*x*) exceeds *n*_Free_(*x*), so, *n*_Trap_(*x*) + *n*_Free_(*x*) ~ *n*_Trap_(*x*), whereas at high *V*_G_s, *n*_Trap_(*x*) is smaller than *n*_Free_(*x*)_,_ so, *n*_Trap_(*x*) + *n*_Free_(*x*) ~ *n*_Free_(*x*). Therefore, at each condition, *E*_IGZO_(*x*) in Eq. (6) can be simplified as Eq. (9) and (10) when *n*_Trap_(*x*) >  > *n*_Free_(*x*) and *n*_Trap_(*x*) <  < *n*_Free_(*x*), respectively.

9 $$E_{IGZO} \left( x \right) \approx \sqrt {\frac{{2N_{T} \left( {kT_{T} } \right)^{2} }}{{\varepsilon_{S} }}\exp \left( {\frac{{E_{F0} - E_{C} + q\varphi_{s} - qV_{CH} }}{{kT_{T} }}} \right)} ,\,\,\,\left( {n_{Trap} \left( x \right) \gg n_{Free} \left( x \right)} \right)$$

10 $$E_{IGZO} \left( x \right) \approx \sqrt {\frac{{2N_{C} kT}}{{\varepsilon_{S} }}\exp \left( {\frac{{E_{F0} - E_{C} + q\varphi_{s} - qV_{CH} }}{kT}} \right)} ,\,\,\,\left( {n_{Trap} \left( x \right) \ll n_{Free} \left( x \right)} \right)$$

Considering that *I*_D_ in IGZO TFTs can be expressed as^27^

11 $$I_{D} = q\frac{W}{L}\mu_{FE} \int\limits_{0}^{{V_{D} }} {\int\limits_{0}^{{t_{S} }} {n_{Free} \left( {\varphi ,V_{CH} } \right)dx} dV_{CH} } = q\frac{W}{L}\mu_{FE} \int\limits_{0}^{{V_{D} }} {\int\limits_{0}^{{\varphi_{S} }} {\frac{{n_{Free} \left( {\varphi ,V_{CH} } \right)}}{{E_{IGZO} \left( x \right)}}d\varphi } dV_{CH} }$$

*I*_D_ can be simplified under the conditions of *n*_Trap_(*x*) >  > *n*_Free_(*x*) and *n*_Trap_(*x*) <  < *n*_Free_(*x*), respectively, as follows:

12 $$\begin{gathered} I_{{D1}} = q\frac{W}{L}\mu _{{FE}} \frac{{\sqrt {\varepsilon _{S} } N_{C} }}{{\sqrt {2N_{T} \left( {kT_{T} } \right)^{2} } }}\exp \left( {\left( {E_{{F0}} - E_{C} + q\varphi _{S} } \right)\left( {\frac{1}{{kT}} - \frac{1}{{2kT_{T} }}} \right)} \right)\left( {\frac{1}{{kT}} - \frac{1}{{2kT_{T} }}} \right)^{{ - 2}} \hfill \\ \left( {1 - \exp \left( { - qV_{D} \left( {\frac{1}{{kT}} - \frac{1}{{2kT_{T} }}} \right)} \right)} \right) \approx q\frac{W}{L}\mu _{{FE}} \frac{{\sqrt {\varepsilon _{S} } N_{C} }}{{\sqrt {2N_{T} \left( {kT_{T} } \right)^{2} } }}\exp \left( {\left( {E_{{F0}} - E_{C} + q\varphi _{S} } \right)\left( {\frac{1}{{kT}} - \frac{1}{{2kT_{T} }}} \right)} \right)\left( {\frac{1}{{kT}} - \frac{1}{{2kT_{T} }}} \right)^{{ - 2}} ,{\text{ }} \hfill \\ {\text{ }}V_{D} > 3kT,\,\,\,\,\,\,\left( {n_{{Trap}} \left( x \right) \gg n_{{Free}} \left( x \right)} \right) \hfill \\ \end{gathered}$$

13 $$\begin{aligned} I_{{D2}} & = q\frac{W}{L}\mu _{{FE}} \int\limits_{0}^{{V_{D} }} {\sqrt {2\varepsilon _{S} N_{C} kT\exp \left( {\frac{{E_{{F0}} - E_{C} + q\varphi _{S} - qV_{{CH}} }}{{kT}}} \right)} dV_{{CH}} } \\ & = q\frac{W}{L}\mu _{{FE}} \int\limits_{0}^{{V_{D} }} {C_{{OX}} \left( {V_{G} - V_{{FB}} - \varphi _{S} - V_{{CH}} } \right)dV_{{CH}} } \\ & = q\frac{W}{L}\mu _{{FE}} C_{{OX}} \left( {V_{G} - V_{{FB}} - \varphi _{S} - \frac{1}{2}V_{D} } \right)V_{D} ,\,\,\,\,\,\,\left( {n_{{Trap}} \left( x \right) \ll n_{{Free}} \left( x \right)} \right) \\ \end{aligned}$$

where, the boundary conditions for *V*_CH_ are *V*_CH_(y = 0) = 0 and *V*_CH_(y = *L*) = *V*_D_, respectively. In Eqs. (12) and (13), *μ*_FE_ (= *P*_TLC_μ_Per_) is the field-effect mobility determined based on the trap-limited and percolation conduction mechanisms, where the effect of trap-limited conduction can be considered as ratio of *n*_Free_ and *n*_Trap_, yielding *P*_TLC_ = *n*_Free_/(*n*_Free_ + *n*_Trap_). *μ*_Per_ is the percolation mobility of^28^

14 $$\mu_{per} = \mu_{0} \exp \left( { - \frac{{q\left( {\phi_{B0} - \Delta \phi_{B} } \right)}}{kT} + \frac{{\left( {q\sigma_{B0} } \right)^{2} }}{{2\left( {kT} \right)^{2} }}} \right) = \mu_{0} *\exp \left( {\frac{{\gamma_{B} \Delta E_{F} }}{kT}q\phi_{B0} } \right),$$

where *μ*_0_* = *μ*_0_exp[-*qϕ*_B0_/*kT* + (*qσ*_B0_)^2^/(*kT*)^2^] is an effective band mobility. Here, percolation conduction, governed by potential barriers located above *E*_C_, can be modeled as a mobility scaled from the intrinsic band mobility (*μ*_0_), under the assumption of a Gaussian random distribution of potential barriers characterized by a mean value (*φ*_B0_) and variance (*σ*_B0_). *φ*_B0_ can be lowered by Δ*φ*_B0_ due to thermally excited electrons, which is influenced by the shift in the Fermi level (Δ*E*_F_). The reduction factor γ_B_ is defined as *γ*_B_ = (*D*_B_ − *W*_B_)/*D*_B_, where *D*_B_ and *W*_B_ represent the spatial distance and width of the potential barriers in IGZO, respectively. Equations (12) and (13) can be combined as a total *I*_D_ using a harmonic average^29^

15 $$I_{D} = \left( {\frac{1}{{\left( {I_{D1} } \right)^{m} }} + \frac{1}{{\left( {I_{D2} } \right)^{m} }}} \right)^{{ - \frac{1}{m}}} ,$$

where *m* is the fitting coefficient.

**Fig. 4:**
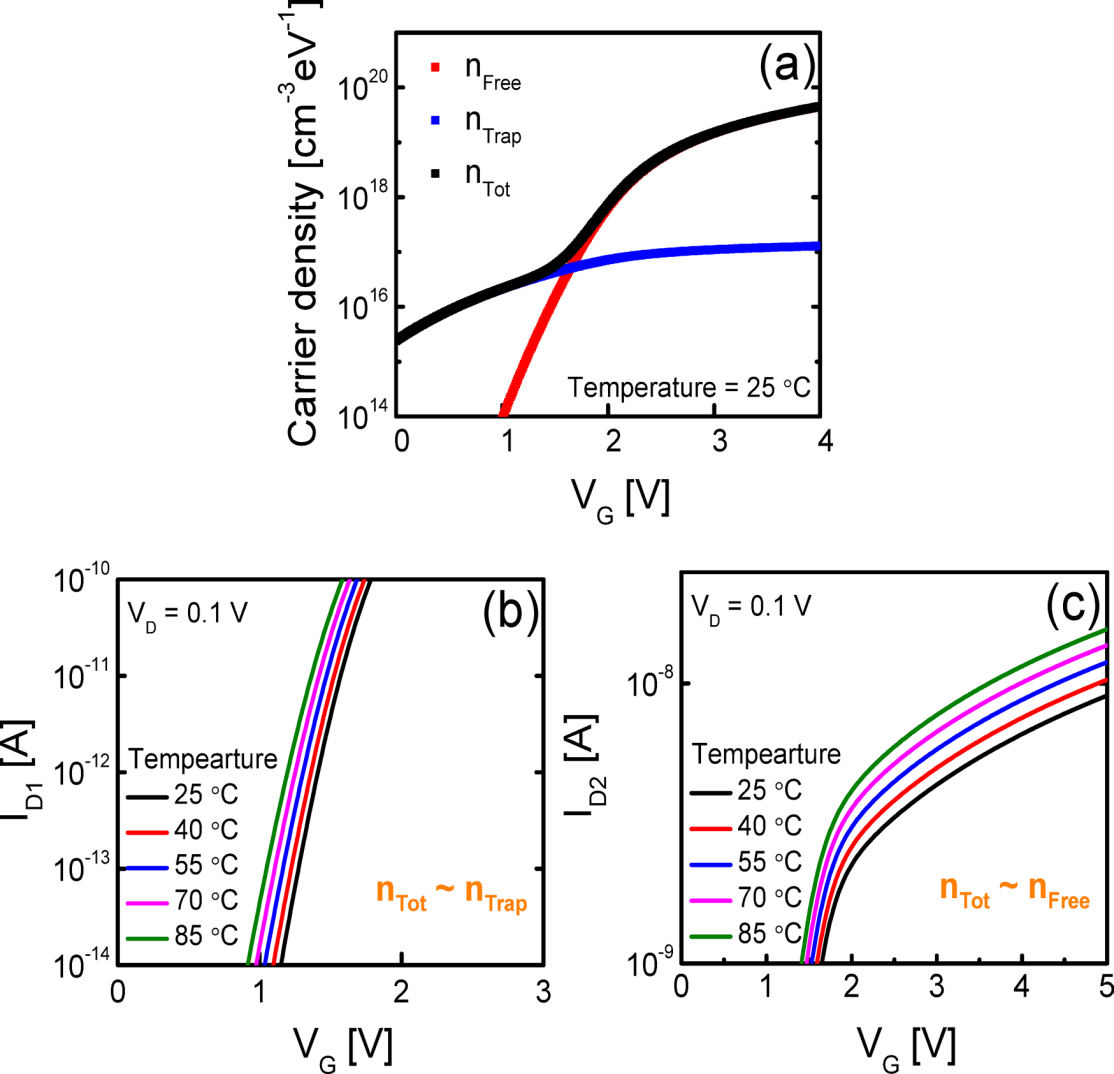
(**a**) *n*_Free_, *n*_Trap_, and *n*_Tot_ (= *n*_Free_ + *n*_Trap_) calculated as a function of *V*_G_ at 25 °C. Transfer curves calculated using the derived model at different temperatures ranging from 25 to 85 °C when (**b**) *n*_Tot_ ~ *n*_Trap_ and (**c**) *n*_Tot_ ~ *n*_Free_.

Figure 4a shows the *n*_Free_, *n*_Trap_, and *n*_Tot_ (= *n*_Free_ + *n*_Trap_) calculated as a function of *V*_G_ using Eqs. (3) and (4) at 25 °C, and Figs. 4b and c show the transfer curves calculated using Eqs. (12) and (13) at different temperatures ranging from 25 °C to 85 °C when *n*_Tot_ ~ *n*_Trap_ (Fig. 4b) and *n*_Tot_ ~ *n*_Free_ (Fig. 4c), respectively. The model parameters used to calculate *n*_Free_, *n*_Trap_ and each transfer curves are shown in Table 1. From the calculation results in Figs. 4b and c, it is observed that when *n*_Tot_ ~ *n*_Trap_ (at low *V*_G_s), the *V*_G_ range required to achieve the same *I*_D_ variation increases with temperature, whereas when *n*_Tot_ ~ *n*_Free_ (at high *V*_G_s), the required *V*_G_ range decreases as temperature increases.

**Table 1:** Model parameters used to calculate *n*_Free_, *n*_Trap_, and transfer curves of the IGZO TFTs in this study.

Parameter	Quantity	value
*W*/*L*	Channel width/channel length	5
*ε*_OX_	Permittivity of gate insulator	4
*t*_OX_	Thickness of Insulator	120 × 10^–7^ cm
*N*_C_	Effective density of states of free carriers	5 × 10^18^ cm^−3^
*ε*_S_	Permittivity of IGZO	10
*μ*_band_*	Band mobility	20 cm^2^/Vs
*E*_F0 _− *E*_C_	Activation energy at flat band conditions	− 0.3 eV
*N*_T_	Density of trap states extrapolated to E_C_	5 × 10^17^ cm^−3^ eV^−1^
*kT*_T_	Characteristic energy for acceptor-like tail states	0.19 eV
*γ*_B_*φ*_B0_	Spatial coherence ratio of the potential barriers	0.004 eV
*m*	fitting coefficient	1

**Fig. 5:**
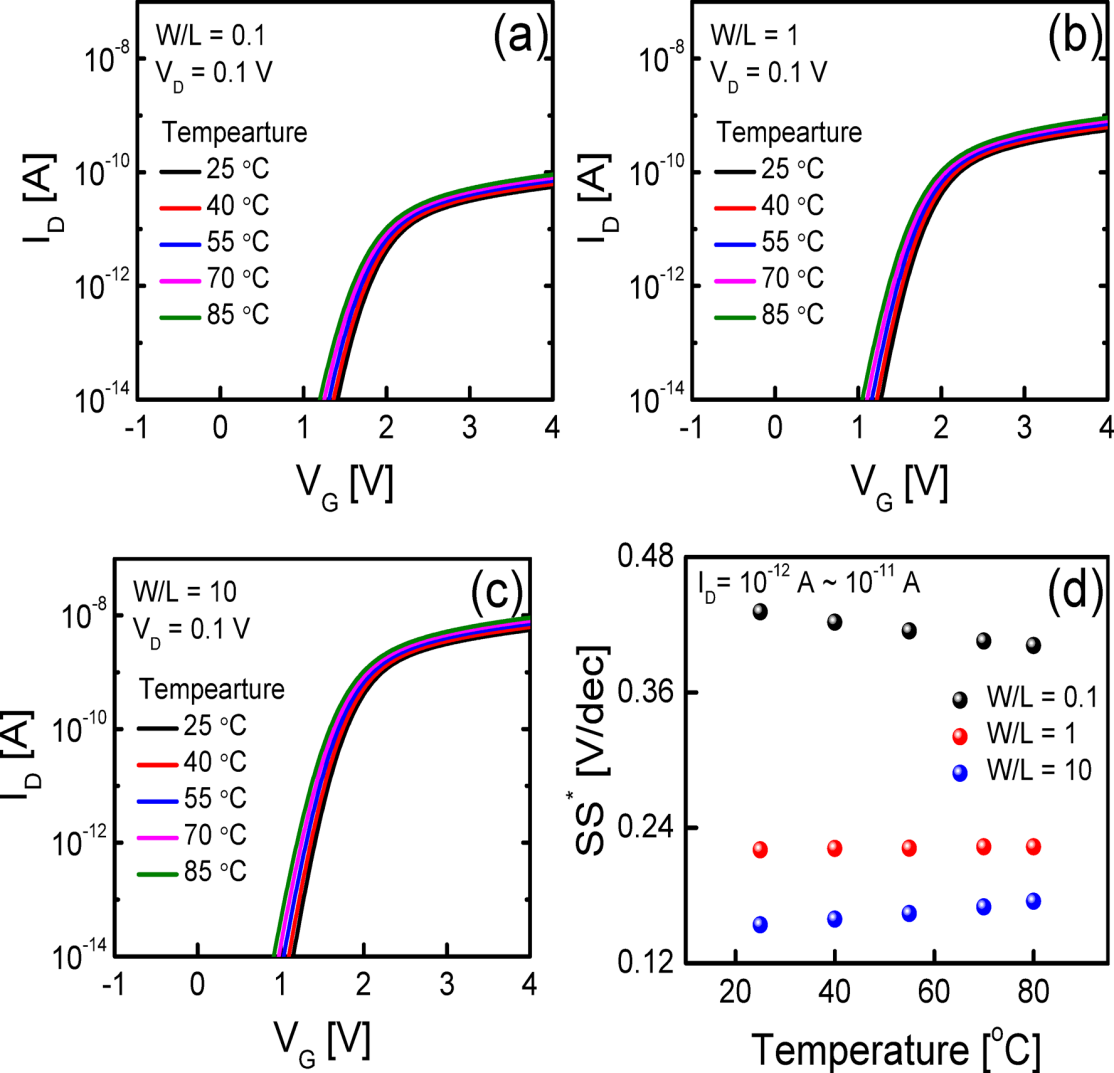
Full range transfer characteristics calculated using the derived model for IGZO TFTs with *W*/*L* ratios of (**a**) 0.1, (**b**) 1, and (**c**) 10, over a temperature range of 25 °C to 85 °C. (**d**) Extracted SS^*^ values for each IGZO TFT at various temperatures within the same range.

Figures 5a–c show the transfer curves calculated using Eq. (15) at temperatures ranging from 25 to 85 ^o^C for IGZO TFTs with *W*/*L* = 0.1 (Fig. 5a), 1 (Fig. 5b), and 10 (Fig. 5c), respectively. Figure 5d summarizes the SS^*^ values extracted from each IGZO TFT at various temperatures within the same range. It is observed in Fig. 5d that the SS^*^ value increases with increasing temperature for *W*/*L* = 10, whereas it decreases with increasing temperature for *W*/*L* = 0.1. Based on the calculation results shown in Fig. 4, this behavior can be attributed to the fact that, for the device with *W*/*L* = 10, the *I*_D_ region used to extract the SS^*^ value satisfies the *n*_Tot_ ~ *n*_Trap_ condition, while for the device with *W*/*L* = 0.1, the same current range satisfies the *n*_Tot_ ~ *n*_Free_ condition. The calculation results presented in Fig. 5 exhibit a consistent trend with the experimental results shown in Fig. 2, where the SS^*^ values obtained from fabricated IGZO TFTs with *L* = 5 and 7 μm increase with increasing temperature, whereas the value obtained from the IGZO TFT with *L* = 10 μm decreases as the temperature increases.

**Fig. 6:**
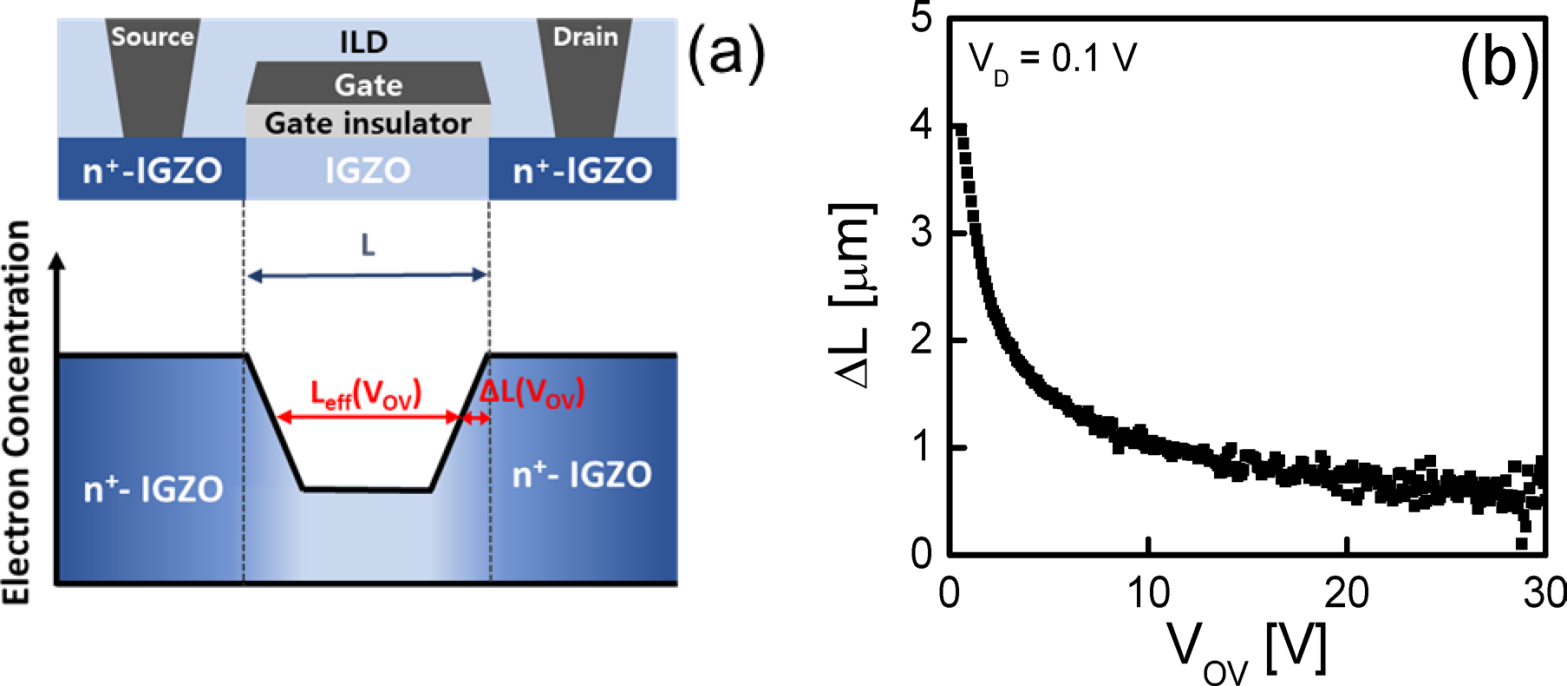
(**a**) Schematic plot illustrating the concept of *L*_eff_(*V*_OV_) in SA TG coplanar IGZO TFTs. (**b**) Δ*L*(*V*_OV_) (*L*_eff_(V_OV_) = *L* − Δ*L*(*V*_OV_)) extracted from the fabricated SA TG coplanar IGZO TFTs using the paired *V*_G_-based transmission line method.

The results in Figs. 4 and 5 demonstrate that the variation in the temperature dependence of the SS^*^ with *L*, observed in Fig. 2, originates from differences in the dominant type of electrons within the *I*_D_ range used to extract the SS^*^, depending on *L*. Nevertheless, the significant change in the temperature dependence of the SS^*^, despite only a twofold increase in *L* from 5 to 10 μm in Fig. 2, cannot be fully explained by this interpretation alone. To better understand the phenomenon observed in Fig. 2, we compared the effective channel lengths (*L*_eff_s) of the fabricated SA TG coplanar IGZO TFTs. As stated, prior research reported that oxygen vacancies or hydrogen atoms diffused from the n^+^-doped source/drain extension regions into the IGZO channel layer during the subsequent annealing process, leading to a reduction in *L*_eff_ in the SA TG coplanar IGZO TFTs^30,31^. Figures 6a indicates the schematic plot illustrating the concept of *L*_eff_(*V*_OV_) in SA TG coplanar IGZO TFTs, where *V*_OV_ is the overdrive voltage (*V*_OV_ = *V*_G _− *V*_T_) and *V*_T_ is the threshold voltage. Figure 6b shows the Δ*L*(*V*_OV_) (*L*_eff_(*V*_OV_) = *L* − Δ*L*(*V*_OV_)) extracted using the paired *V*_G_-based transmission line method^18,32^. Considering that the SS^*^ values were extracted at very low *V*_OV_s close to 0 V, the *L*_eff_ of the 10 μm TFT appears to be at least 5 to 6 times larger than that of the 5 μm TFT in the *I*_D_ region used to extract the SS^*^ value. This is believed to be the reason for the distinct difference observed in the temperature dependence of the SS^*^ in the fabricated SA TG coplanar IGZO TFTs, despite only a twofold change in *L*.

Generally, in disordered semiconductor-based TFTs, the subthreshold region is defined as the regime where *n*_Tot_ ~ *n*_Trap_, and thus the *SS* is typically extracted from this region^33^. However, in the case of IGZO TFTs, the bandgap of IGZO is very large (greater than 3.0  eV)^34,35^. As a result, the off-currents and small subthreshold currents are often below the detection limit of conventional DC measurement equipment^36^. Consequently, the *I*_D_ range commonly used for extracting *SS* in prior studies for IGZO TFTs (*I*_D_ ~ 10^−13^ to 10^–10^ A) may correspond to different operating regimes such as *n*_Tot_ ~ *n*_Trap_, *n*_Tot_ ~ *n*_Trap_ + *n*_Free_ or *n*_Tot_ ~ *n*_Free_, depending on various factors including the device dimension. This variation is considered to be a major reason for the inconsistent experimental results reported in prior studies regarding the temperature dependence of *SS* in oxide TFTs.

## Conclusion

In this study, we observed that the temperature dependence of SS^*^ varies with *L* in SA TG coplanar IGZO TFTs, and investigated the underlying mechanisms. Experimentally, as the temperature increased from 25 to 85 ^°^C, the SS^*^ increased with temperature for the device with *L* = 5 μm and 7 μm whereas it decreased for devices with *L* = 10 μm. To elucidate the origin of the observed phenomenon, *φ*_S_-based *I*_D_ modeling was performed for IGZO TFTs. The calculation results revealed that in the *n*_Tot_ ~ *n*_Trap_ (at low *V*_G_s), the *V*_G_ range required to achieve the same *I*_D_ variation increases with temperature, whereas in the *n*_Tot_ ~ *n*_Free_ (at high *V*_G_s), the required *V*_G_ range decreases with increasing temperature. Based on the developed model, it was considered that for devices with shorter channel lengths, the SS^*^ was extracted in the *n*_Tot_ ~ *n*_Trap_ region, whereas for devices with longer channel lengths, the measurement region corresponded to the *n*_Tot_ ~ *n*_Free_ region. Furthermore, the lateral diffusion of donor impurities from the source/drain extension regions into the IGZO channel layer in the SA TG coplanar IGZO TFTs is believed to enlarge the difference in *L*_eff_ compared to the drawn gate length differences. This effect amplifies the variation in the temperature dependence of SS^*^ even between devices with relatively small differences in drawn gate lengths.

## Methods

An Al layer was deposited and patterned on a glass substrate to serve as the back gate electrode, which also functions as a light shield. Subsequently, a 300-nm thick SiOx layer was deposited as a buffer layer using plasma-enhanced chemical vapor deposition (PECVD) at 350 °C. A 30-nm thick IGZO channel layer (In:Ga:Zn = 1:1:1 atomic percentage) was then deposited via radio-frequency magnetron sputtering, followed by the deposition of a 120-nm thick SiOx gate insulator using PECVD at 200 °C. A Mo layer was then deposited and patterned to form the gate electrode. After the deposition and patterning of the Mo gate electrode and the SiOx gate insulator, a SiOx and SiNx layer were sequentially deposited as an interlayer dielectric (ILD) using PECVD and patterned to create via holes. The source and drain electrodes, made of Al, were deposited and patterned on the n^+^-doped IGZO source/drain extension regions. In these regions, hydrogen, diffusing from the PECVD-deposited ILD, acts as an electron donor in the IGZO, thus forming the source/drain extension regions. Finally, the TFTs were passivated with a SiO_X_ layer and thermally annealed at 340 °C to achieve stable and uniform electrical characteristics.

## Data Availability

The datasets used and/or analysed during the current study available from the corresponding author on reasonable request.
